# Bacterial persisters in long-term infection: Emergence and fitness in a complex host environment

**DOI:** 10.1371/journal.ppat.1009112

**Published:** 2020-12-14

**Authors:** Jennifer A. Bartell, David R. Cameron, Biljana Mojsoska, Janus Anders Juul Haagensen, Tacjana Pressler, Lea M. Sommer, Kim Lewis, Søren Molin, Helle Krogh Johansen

**Affiliations:** 1 The Novo Nordisk Foundation Center for Biosustainability, Technical University of Denmark, Kgs. Lyngby, Denmark; 2 Antimicrobial Discovery Center, Department of Biology, Northeastern University, Boston, Massachusetts, United States of America; 3 Department of Intensive Care Medicine, Inselspital, Bern University Hospital, University of Bern, Bern, Switzerland; 4 Department of Clinical Microbiology, Rigshospitalet, Copenhagen, Denmark; 5 Cystic Fibrosis Center, Rigshospitalet, Copenhagen, Denmark; 6 Department of Clinical Medicine, University of Copenhagen, Copenhagen, Denmark; University of North Carolina at Chapel Hil, UNITED STATES

## Abstract

Despite intensive antibiotic treatment, *Pseudomonas aeruginosa* often persists in the airways of cystic fibrosis (CF) patients for decades, and can do so without antibiotic resistance development. Using high-throughput screening assays of bacterial survival after treatment with high concentrations of ciprofloxacin, we have determined the prevalence of persisters in a large patient cohort using 460 longitudinal isolates of *P*. *aeruginosa* from 39 CF patients. Isolates were classed as high persister variants (Hip) if they regrew following antibiotic treatment in at least 75% of the experimental replicates. Strain genomic data, isolate phenotyping, and patient treatment records were integrated in a lineage-based analysis of persister formation and clinical impact. In total, 19% of the isolates were classified as Hip and Hip emergence increased over lineage colonization time within 22 Hip+ patients. Most Hip+ lineages produced multiple Hip isolates, but few Hip+ lineages were dominated by Hip. While we observed no strong signal of adaptive genetic convergence within Hip isolates, they generally emerged in parallel or following the development of ciprofloxacin resistance and slowed growth. Transient lineages were majority Hip-, while strains that persisted over a clinically diagnosed ‘eradication’ period were majority Hip+. Patients received indistinguishable treatment regimens before Hip emergence, but Hip+ patients overall were treated significantly more than Hip- patients, signaling repeated treatment failure. When subjected to *in vivo*-similar antibiotic dosing, a Hip isolate survived better than a non-Hip in a structured biofilm environment. In sum, the Hip phenotype appears to substantially contribute to long-term establishment of a lineage in the CF lung environment. Our results argue against the existence of a single dominant molecular mechanism underlying bacterial antibiotic persistence. We instead show that many routes, both phenotypic and genetic, are available for persister formation and consequent increases in strain fitness and treatment failure in CF airways.

## Author summary

The persister phenotype, the ability of bacterial cells to survive antibiotic treatment without development of antibiotic resistance, is hypothesized to be an important contributor to treatment failure in recurrent bacterial infections. Using isolates of the bacterial pathogen *Pseudomonas aeruginosa* collected over a decade from the airways of 39 young cystic fibrosis patients, we investigated the emergence, continuity, and contribution to fitness of the persister phenotype in a clinical scenario with high levels of antibiotic treatment. We observe high-persister variants in 56% of the patients, but no signal of adaptive genetic convergence supporting their appearance, and no difference in patient treatment regimens before variant emergence. However, bacterial lineages (distinct bacterial strains infecting a patient over time) producing high-persister variants also produce isolates with antibiotic resistance and/or slowed growth rate. These lineages are also significantly less likely to be transient and more likely to persist in patient lungs over long periods of time without detection in the clinic. In sum, we conclude that the persister phenotype can emerge by many adaptive routes and offers important fitness contributions in the complex *in vivo* environment of cystic fibrosis airways.

## Introduction

Antibiotic-tolerant persister cells are suspected to be a significant clinical problem. Compared with antibiotic-resistant bacteria, far less is understood about the contribution of persisters to treatment failure, even though persisters were in fact described shortly after the clinical introduction of antibiotics [1]. Persisters are distinct from antibiotic-resistant mutants, as they do not grow in the presence of antibiotics. Instead, they survive during antibiotic exposure but retain the capacity to resuscitate and restore the population when antibiotic concentrations drop [2–4]. However, our understanding of the physiology and clinical relevance of persister cells is limited, given the difficulty in reliably isolating what is theorized to be a stochastic phenotype *in vitro*, much less monitoring this phenotype in routine clinical care. Thus, while a few characterizations of small environmental isolate collections have shown that formation of persisters varies across strains [5–8], few studies have assayed persister formation in clinical or other complex environmental scenarios. One study of oral carriage (0–19 weeks) of *Candida albicans* isolates from 22 cancer patients undergoing chemotherapy found that patients with carriage of greater than 8 weeks had significantly higher persister levels than those with less than 8 weeks of carriage, but did not address the underlying mechanisms of persistence for this pathogen [9]. To examine the underpinnings and long-term impact of the high-persister phenotype in a clinical scenario, both a large, aligned patient cohort that places the bacteria under similar environmental stresses as well as isolate sampling at a resolution that captures the emergence and longevity of the phenotype are needed.

*P*. *aeruginosa* is the most frequent cause of chronic airway infections in patients with CF [10,11]. Mutations in the cystic fibrosis transmembrane conductance regulator (*CFTR*) gene often result in inefficient mucociliary clearance of bacteria from the airways, creating opportunities for bacterial colonization [12,13]. Upon entering the host, environmental *P*. *aeruginosa* adapts to the CF lung environment, ultimately establishing an incurable airway infection [14,15]. Despite intensive antibiotic treatment from the first discovery of the bacterium in the lung, resistance emergence in the first years of infection is surprisingly low [16,17]. In the absence of clinically defined antibiotic resistance, survival of the bacteria is likely enabled by diverse and often co-occurring traits including slowed growth rate, biofilm formation, and the production of antibiotic tolerant persister populations [18–20]. How persister cells interrelate with other co-selected changes (e.g. slowed growth rate, biofilm formation), and the additional contribution of host factors (e.g. the immune system) is rarely accounted for in *in vitro* persister studies, but is likely clinically important.

Co-evolving traits also complicate the search for genetic mechanisms of the high-persister phenotype that are clinically impactful. While persister cells are stochastic phenotypic variants in any bacterial population, genetic changes in bacterial populations have been shown to produce a high persister state, producing increased numbers of antibiotic tolerant cells following exposure to antibiotics in *in vitro* studies of pathogenic species [21,22]. Some of these genetic changes have also been observed in clinical isolates; within a set of 477 commensal or urinary tract infection isolates of *Escherichia coli*, 24 exhibited a mutation in the canonical persister gene *hipA*, and the causality between a *hipA7* mutation and a Hip phenotype confirmed by deleting this allele from one of the clinical isolates [23]. An investigation in young CF patients showed an increase in high-persister phenotype in early/late infection isolate pairs from 14 patients. In this study, 35 longitudinal *P*. *aeruginosa* isolates taken from one child over a 96-month period showed increased levels of persister cells over time as well as an accumulation of 68 mutations between the first and last isolate [18]. However, the mutations in the single patient resembled those known to accumulate in other CF patients over infection rather than any mutations previously associated with the Hip phenotype in persister-focused *in vitro* studies (S1 Table).

To acquire a high-resolution pan-cohort perspective of high-persister emergence, genetic mechanism, and impact in long-term infections, we have screened 460 longitudinal isolates of *P*. *aeruginosa* collected from 39 young CF patients over a 10-year period from early colonization onward for high-persister variants (Hip, defined by survival of at least 75% of replicates) tolerant to the antibiotic ciprofloxacin. This unique isolate collection allows us to determine Hip prevalence and dynamics during each colonizing strain’s transition from environmental isolate to persistent pathogen. We describe relationships between the Hip phenotype and response to each drug, the age of the isolate, and other adaptive traits in longitudinal infections. We show that the Hip phenotype, defined in this study as a strong and reliable recovery from antibiotic challenge that is a serious concern for the clinic, is a widespread trait. We further search for genetic and phenotypic changes associated with the Hip phenotype in independent clonal lineages within distinct patients, which may suggest adaptive routes to producing this phenotype. Finally, we show that the Hip phenotype generally accumulates over time in patients via several archetypal patterns, appears to contribute to long-term persistence of lineages despite high levels of antibiotic treatment, and increases the fitness of colonizing populations of *P*. *aeruginosa* in antibiotic-treated CF patient lungs.

## Results

### The isolate collection

We examined a collection of 460 *P*. *aeruginosa* airway isolates obtained from 39 young CF patients over a 10 year period while they were treated at the Copenhagen CF Centre at Rigshospitalet [24]. These patients represent a cohort aligned at the early infection stage and undergoing similar treatment regimens per CF Centre guidelines, with repeated culture of *P*. *aeruginosa* from their monthly sputum sampling within a time frame of 2–10 years. Patient inclusion was on a rolling basis over the study period in order to capture all early colonization cases. This resulted in a median patient age of 8.11 (age range: 1.44–24.14) at their first isolate included this collection, and a median of 11 isolates per patient (range: 2–28) collected over a median of 4.9 years (range 0.17–10.18). Early isolates therefore represent bacteria that have not been exposed to decades of antibiotic treatment (a usual concern in CF isolate collections from adults) before the study start excepting rare cases of strain transmission from another patient.

The bacterial CF isolates have been grouped into 52 genetically distinct clone types [24], and while many patients retained a monoclonal infection during the entire course of infection, half (n = 20, 51.3%) were infected at least transiently with another clone type. To effectively account for these multi-clonal infections, clinical isolates are described by their patient-specific lineage combining the clone type and the patient of origin (74 lineages in total). Throughout this paper, we will also refer to ‘Time since first detection’ for each isolate, which represents the length of time between first detection and subsequent isolations of the same patient-specific lineage.

### Identification of high-persister (Hip) isolates by high-throughput screening

**Fig 1:**
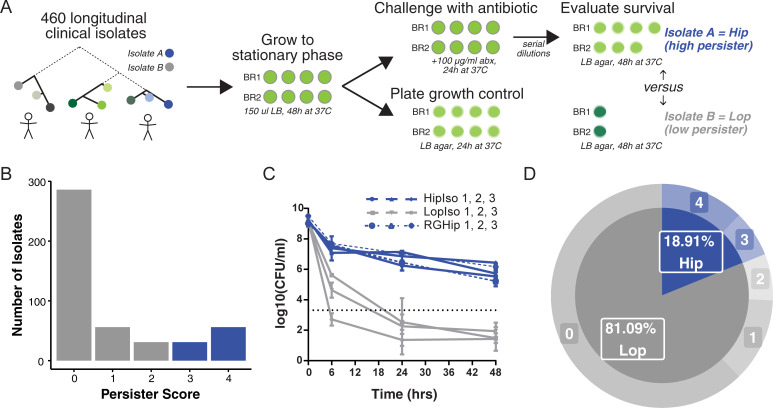
High-throughput screening approach for isolates with a high persister (Hip) phenotype. **(A)** A large collection of *Pseudomonas aeruginosa* clinical isolates were grown to stationary phase in quadruplicate wells for two biological replicate (BR) experiments (each isolate tested 8 times in total). Each isolate was treated with 100 μg/ml of ciprofloxacin for 24 hours, while growth was assessed by plating on LB agar. Following antibiotic treatment, cultures were diluted then plated on agar, at which point survival was assessed. Each isolate was given a persister score based on consistent replicate survival following treatment. Isolates for which 3–4 replicates survived for each BR were given a score of 3–4, respectively, and were considered high persisters (Hip). Isolates with a respective score of 0–2 were considered low persisters (Lop). **(B)** Score distribution of *P*. *aeruginosa* Hip (blue) and Lop (grey) isolates against ciprofloxacin. **(C)** Traditional time-kill assays were performed for three Hip (HipIso 1–3, solid blue lines) and three Lop isolates (LopIso 1–3, solid gray lines) from the same patient to validate the high throughput screen. Colony forming units (CFU) per ml were determined following treatment with 100 μg/ml of ciprofloxacin. Data are the mean of 3 independent biological cultures, bars represent SD. The dashed black line represents the limit of detection. Three independent ciprofloxacin-exposed colonies were regrown from each of the residual cultures of the persister assays for the Hip strains and then treated with the same persister assay method (RGHip 1–3, blue dashed lines), showing the same killing kinetics as the first assay. **(D)** We present the final distribution of Hip versus Lop isolates via pie chart.

We screened the collection of *P*. *aeruginosa* isolates for the propensity to survive in the presence of high concentrations of antibiotics. We chose the fluoroquinolone antibiotic ciprofloxacin because it is frequently used to treat early *P*. *aeruginosa* infections in CF patients and is bactericidal toward stationary phase *P*. *aeruginosa* as it targets DNA gyrase [25,26]. Briefly, *P*. *aeruginosa* subcultures in micro-titer plates were grown for 48 hours until they reached stationary phase, after which they were challenged with ciprofloxacin (100 μg/ml) for 24 hours before survival was assessed (Fig 1A). This antibiotic concentration was orders of magnitude above the 0.5 μg/ml resistance breakpoint from the European Committee on Antimicrobial Susceptibility Testing (EUCAST), minimising the chance that the screen selected for isolates with modestly elevated minimum inhibitory concentrations (MICs). Isolates were assayed eight times (technical quadruplicates performed in duplicate biological experiments with a positive growth control for at least 3 of 4 replicates in each experiment) and scored based on the capacity to re-grow after antibiotic treatment. An isolate was given a score of 0 if it failed to re-grow in any replicate of an experiment, a score of 1 if it grew once in both biological duplicates, a score of 2 if it grew in half of the technical replicates in each experiment, a score of 3 if it grew in at least three replicates in each experiment, and a score of 4 if it grew in all replicates (Fig 1B).

We defined high-persister (Hip) isolates as those scoring either 3 or 4 (i.e. at least six of eight technical replicates re-grew) and low persister (Lop) isolates as those scoring between 0 and 2. This stringent scoring system was used to minimize the mis-classification of false Hips and focus our analysis on isolates reliably producing high levels of persister cells, representing the most concerning phenotype in a clinical environment. Isolates with a score of 4 made up the largest Hip group, while the largest Lop group consisted of isolates scored as 0, failing to grow in any replicate (Fig 1B). To validate this classification system, we selected six isolates from the same patients, three of which were putative Hips, and three of which were classed as Lops and performed (i) time-dependent killing assays, (ii) concentration-dependent killing assays, and (iii) persister regrowth killing assays. The isolates displayed typical killing kinetics, with a ‘persister plateau’ observable following 24 hours of treatment (Fig 1C). Each of the three putative Hip strains harbored a greater subpopulation of surviving persister cells (>4 log_10_CFU/ml), and none of the Lop strains reached the detection limit of the high throughput screen after 24 hours (3.3 log_10_CFU/ml, dotted black line) when examined using the time-dependent killing assay. Each of the three Hip strains produced the same number of surviving persister cells following 24 hours of ciprofloxacin treatment at both 10μg/ml and 100μg/ml (S1 Fig). Finally, for each putative Hip, three independent colonies formed on agar plates following treatment (100 μg/ml) were regrown and re-exposed to ciprofloxacin. The killing kinetics were similar for the regrown persisters, as for the original isolates (Fig 1C, RGHips). Taken together, the isolates identified using the high-throughput screen are those that produce high numbers of persister cells and thus represent true Hips as defined by recent guidelines [27].

To further validate our high-throughput screening approach, we selected 19 additional isolates, and enumerated CFUs following 24 hours of ciprofloxacin treatment using standard survival assays. Isolates were taken from nine different patients, which represented nine distinct lineages, and spanned different ciprofloxacin MICs (from 0.023 to 4μg/ml). The laboratory strain PAO1, which scored 1 in the high-throughput screen, was included in the validation (n = 26 strains in total). We observed a significant positive correlation between persister score and CFU/ml following treatment (r^2^ 0.5719, p < 0.0001, S2 Fig), thus demonstrating the validity of our experimental approach. In total, 18.9% of the screened isolates (87 isolates) exhibited a persister phenotype (Fig 1D).

### The persister phenotype adapts alongside resistance and reduced growth rate

**Fig 2:**
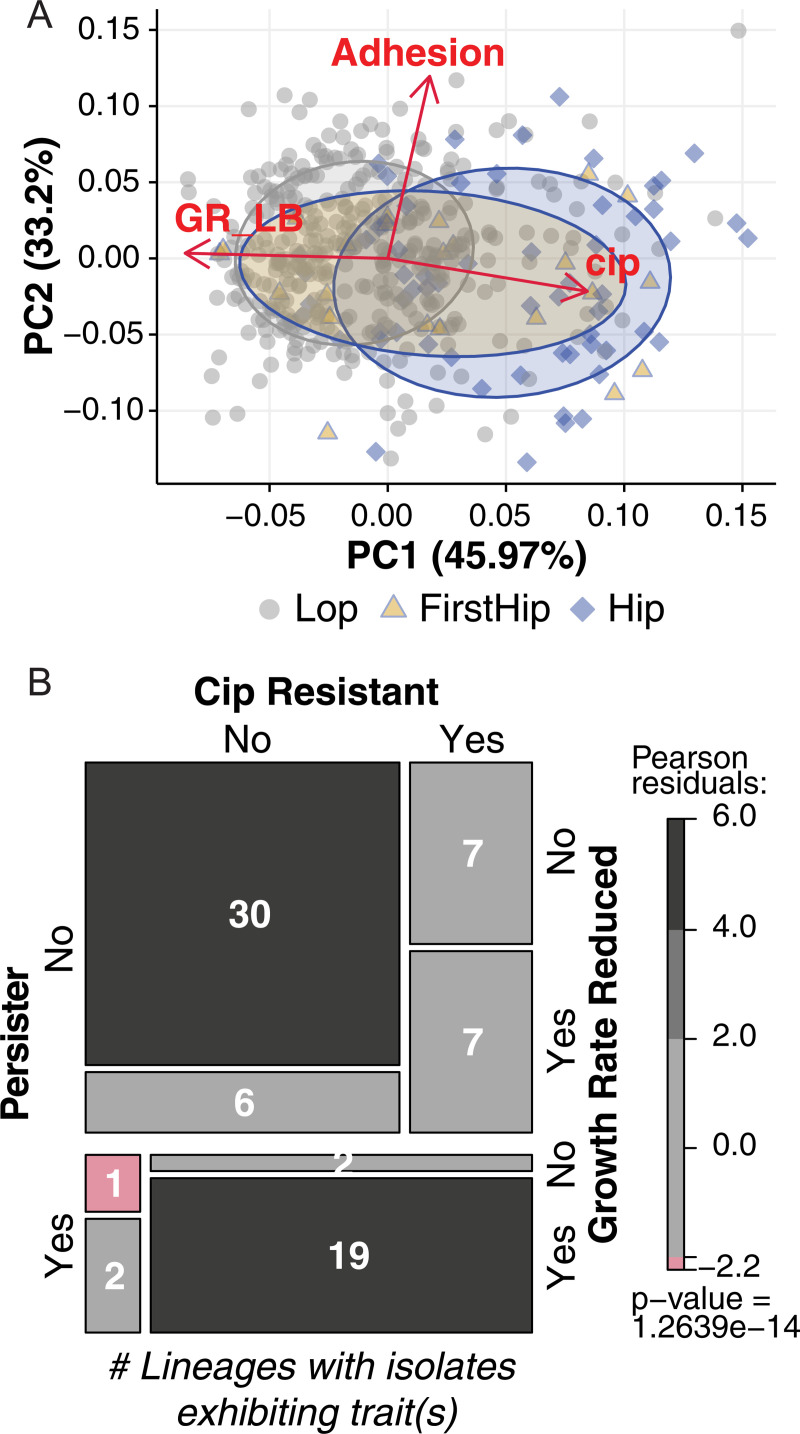
High-persisters in the multi-trait landscape. **(A)** Lop (grey circle) and Hip (blue diamonds were analyzed via principle component analysis with respect to their similarity with other infection-linked traits: growth rate (GR_LB), adhesion, and ciprofloxacin MIC (cip). 446 isolates with complete trait sets were included. Hip isolates do not consistently cluster with any one additional trait. Each symbol represents a *P*. *aeruginosa* isolate. The first Hip isolates from a lineage (FirstHip, yellow triangles) were highlighted as Hip variants with mitigated effects of other accumulating mutations within the lineage to improve cross-lineage comparison. In each case, FirstHip and the remaining Hip isolates shift to various degrees from ‘naïve’ towards ‘adapted’ levels given the particular Hip dataset. We illustrate this using data ellipse enclosing samples approximately within the first standard deviation (t distribution, 68% of the set) for isolate sets characterized as FirstHip (yellow ellipse), and the remaining Hips (blue ellipse). **(B)** We visualized the association between lineages that produced Hips versus resistant isolates and/or slow growing isolates (identified by the minimum growth rate of lineage isolates falling below 70% of the *P*. *aeruginosa* PAO1 growth rate based on a 45 minute generation time in LB in microtiter wells). Association between variables is illustrated by a mosaic plot (multi-way contingency table visualization) where color indicates significant deviation from the expected frequency of lineages in each cell under trait independence using Pearson’s chi-squared test. Presence of isolates with a given trait in a lineage is indicated by ‘Yes’, absence by ‘No’.

The high-persister phenotype in our collection is not arising in isolation from other adaptations. We and others have previously observed that CF isolates adapt towards slow growth rates and increased resistance to antibiotics, and some lineages develop towards a biofilm lifestyle [20,28,29]. Supporting a theory that persistence enables resistance, studies have observed the emergence of persister isolates before resistant isolates *in vitro* [8,30], with a recent study showing this relationship *in vivo* for two bacteremia patients infected by *Staphylococcus aureus* [31]. A specific association between slowing growth rate and the Hip phenotype has also been proposed [32]. To broadly survey overlap with other phenotypes, we used a principal component analysis to evaluate the distribution of Hip (blue diamonds) versus Lop (grey circles) variants by multiple traits under selection pressure in the CF lung using previously published phenotype data for most isolates (described further in Materials and Methods) [20]. We see that Hip variants group with isolates exhibiting more adapted traits (increased antibiotic MICs and slowing growth), but they also appear across the full phenotypic space alongside Lop isolates (Fig 2). We further specifically identified the first Hip variant (FirstHip–blue ellipse with yellow fill) of each lineage (a clone type infecting a given patient) in an attempt to assess these isolates’ other traits and state of adaptation at first appearance, when the accumulated impact of co-evolving traits is lowest on the Hip isolates. We see that FirstHips overlap substantially with both Lops and Hips. This variation of initial adaptive state could be due to different adaptive trajectories with patients as well as lapses of time between Hip emergence and isolation.

Given recent literature on resistance and slowed growth, we specifically compared isolate persister class versus ciprofloxacin resistance as well as slowed growth rate. Thirty-nine percent of the isolates were characterized as resistant to ciprofloxacin based on the EUCAST breakpoint (176 of 451 isolates with available data). Sixty-three of 87 Hip isolates were resistant (72.4%), while 113 of 251 Lops were resistant (31%). Forty percent of isolates showed growth rate reduction (183 of 460 isolates), defined as less than 70% of the growth rate of robustly growing lab strain *P*. *aeruginosa* PAO1. Fifty-nine of 87 Hip isolates were slow growers (87%), whereas 124 of 373 Lops were slow growers (33.2%). These numbers suggest associations between growth rate reduction, resistance, and Hip isolates. However, we intentionally avoid statistical tests for significance as these isolates are affected by lineage bias in both parallel trait adaptation and varying number of isolates cultured for each patient lineage.

Instead, we contrasted the time of emergence of these trait adaptations within each affected lineage both individually and in combination with the date of first Hip isolation (Table 1). We note that our lineage classifications do not guarantee trait continuance within a lineage or co-occurrence within the same isolate, i.e. lineages are capable of producing isolates with trait(s), but individual isolates may not share the set of phenotypes. First, 24 of 74 lineages showed Hip isolate emergence (Hip+ lineages), 35 lineages showed resistant isolate emergence, and 34 lineages showed growth-reduced isolate emergence. Resistant isolates preceded Hip isolates in 8 lineages, emerged simultaneously in 11 lineages, and follow Hip in 2 lineages. With respect to growth rate reduction, we observed that reduction precedes persistence in 9 lineages, emerges simultaneously in 7 lineages, and follows persistence in 5 lineages.

**Table 1:** Emergence of adapted trait isolates versus persister isolates across 74 lineages. Traits assessed included resistance to ciprofloxacin according to the EUCAST breakpoint of 0.5 μg/ml and reduction in growth (isolates which grew slower than 70% of the growth rate of robust lab strain *P*. *aeruginosa* PAO1 grown in similar conditions, i.e. a generation time of greater than 65 minutes). Date of first trait emergence was calculated for the adaptation of each trait, the minimum first date of either trait (in lineages where both adapted) as ‘Either Trait’, and the date by which both traits had adapted (Both Traits). These dates were then compared with the date of first Hip emergence.

Category		# Lineages affected		
	*Both Traits*	*Ciprofloxacin Resistance*	*Growth Rate Reduction*	*Either Trait*
*Trait before persister adaptation*	4	8	9	13
*Simultaneous emergence*	10	11	7	8
*Persister before trait adaptation*	5	2	5	2
*Trait adaptation alone*	7	14	13	20
*Persister adaptation alone*	5	3	3	1

Importantly, we can confirm when comparing data in the ‘Either trait’ versus ‘Both traits’ columns that persister, resistance, and growth reduction traits are only partially overlapping as they evolve. For example, both traits adapt before Hip emergence in 4 lineages, but at least one adapts before Hip emergence in 13 lineages. In contrast, persisters emerge in 5 lineages before at least one of the traits has adapted, but only emerge earlier in 2 lineages if either trait is assessed. Only 7 lineages show emergence of both slowed growth isolates and resistance isolates without Hips (‘Both Traits’), while Hip isolates emerge alone in only 1 lineage (‘Either Trait’). The multi-way mosaic plot of Fig 2A shows presence-absence of isolates with each trait across all lineages (not accounting for emergence time), which emphasizes the significance of this lone persister-only lineage (red). In total, these results emphasize the complexity of selection pressures at play over time on these lineages, resulting in concurrent adaptation of distinct traits that likely influence each other and have related genetic underpinnings.

### Evolution of the persister phenotype is not genetically convergent across patient-specific lineages

Sequencing and identification of genetic variations accumulating within each clone type was performed in a previous study [24] for most of the isolates included in the current screen (403 isolates, 46 lineages). In that previous study, genes targeted in convergent evolution were identified by the significant enrichment of observed lineages with mutations in those genes compared to the number of lineages expected to have mutations in the same genes according to genetic drift (derived from a simulated evolution where lineages accumulate an equivalent number of mutations randomly for 1000 independent evolution simulations) [24]. In our current analysis, we split our dataset into Hip and Lop variants, and then performed this same observed versus expected lineage enrichment analysis for each population (see Materials and Methods for further details). The ratio of lineage enrichment of mutated genes for Hip versus Lop variants allowed us to identify candidate ‘*hip*’ genes for each set (Table 2). For completeness, we also performed an additional genetic analysis focusing on mutations in non-coding sequences (S2 Dataset).

**Table 2:** Lineage-based mutation enrichment analysis. Mutated genes enriched in Hip versus Lop dataset as assessed from a convergent evolution perspective accounting for lineage adaptation. Lineage enrichment ratio was calculated by dividing lineage-based gene mutation enrichment within Hip variants by that within Lop variants for each gene. Top Hip-linked genes were selected via the following criteria: greater than 2 lineages presenting mutations in that gene in the Hip population and a lineage enrichment ratio greater than 2.

Locus	Gene Name	Gene Function	Hip+ Lineage Count	Hip- Lineage Count	Lineage Enrichment Ratio	Total Lineages Hit
PA0339		hypothetical protein	3	2	3.098	3
PA2894		hypothetical protein	4	3	3.062	4
PA4462	rpoN	RNA polymerase sigma-54 factor	3	2	2.985	3
PA4856	retS	RetS (Regulator of Exopolysaccharide and Type III Secretion)	5	5	2.482	5
PA0977		hypothetical protein	3	3	2.395	3
PA3183	zwf	glucose-6-phosphate 1-dehydrogenase	4	4	2.391	4
PA1224		probable NAD(P)H dehydrogenase	3	3	2.186	3
PA1600		probable cytochrome c	3	3	2.179	3
PA2685	vgrG4	VgrG4	3	3	2.099	3
PA3640	dnaE	DNA polymerase III, alpha chain	3	3	2.093	3
PA1380		probable transcriptional regulator	3	3	2.057	3
PA2403	fpvG	FpvG	3	3	2.006	3

In general, searching for *hip* genes accumulating non-synonymous mutations in Hip+ lineages revealed only a weak signal for convergent evolution. Only one lineage assessed in the genetic screen had only Hips present (the only isolate of the lineage assessed in our screen), so practically all lineages with Hip isolates (Hip+ lineages, 29 included in the genetic study) also contained Lops. Thus, mutated genes that were enriched 2–3 fold in independently evolved Hip+ lineages were also frequently present in Lop isolates of the same lineage. Our lineage enrichment ratio ultimately identified 12 mutated genes enriched in ciprofloxacin Hip+ lineages (Table 2).

Of note, there was a surprising lack of the most prominent ‘*hip*’ genes previously identified in *in vitro* studies and screens of *P*. *aeruginosa* (S1 Table). None of the lineage enrichment data pointed toward RNA endonuclease-type toxin-antitoxin systems under adaptive selection, which supports recent research that has questioned the contribution of these systems to persistence in numerous bacterial pathogens [33–35]. Instead, they belonged to diverse functional categories including transcriptional regulation/two component regulatory systems (3 genes), energy metabolism (2 genes) DNA replication and repair (1 gene), and virulence (2 genes, iron uptake and a type VI protein secretion system). Table 2 includes major regulators *rpoN*, known to induce a growth defect when functionally mutated [36], and *retS*, which when functionally mutated induces an array of phenotypic changes linked to chronic infection such as a non-motile biofilm lifestyle [37,38]. *retS* and hypothetical protein PA0977 also overlap with the ‘pathoadaptive’ mutationally enriched gene list identified in our prior study of convergent evolution across all lineages [24].

### Hip variants emerge via diverse incidence patterns

The lack of strong genetic signatures differentiating Hip from Lop isolates motivated us to examine the temporal dynamics of high persister incidence from a lineage perspective, where each strain (clone-type) infecting each patient is classed as an independent lineage. In half of the patients, the earliest *P*. *aeruginosa* isolate in our collection is also the first-ever identified *P*. *aeruginosa* in the clinic and the other patients’ isolates also cover most of the initial colonization phase. We can thus estimate the emergence of the Hip phenotype as *P*. *aeruginosa* adapts from a wild type-similar naïve state into an adapted persistent pathogen. Previous findings have indicated that the number of Hip variants from a lineage may increase over time as the bacteria adapt to the antibiotic pressure in the host, and that once a Hip isolate is observed, it is assumed to persist in the infecting population of the patient [9,18].

**Fig 3:**
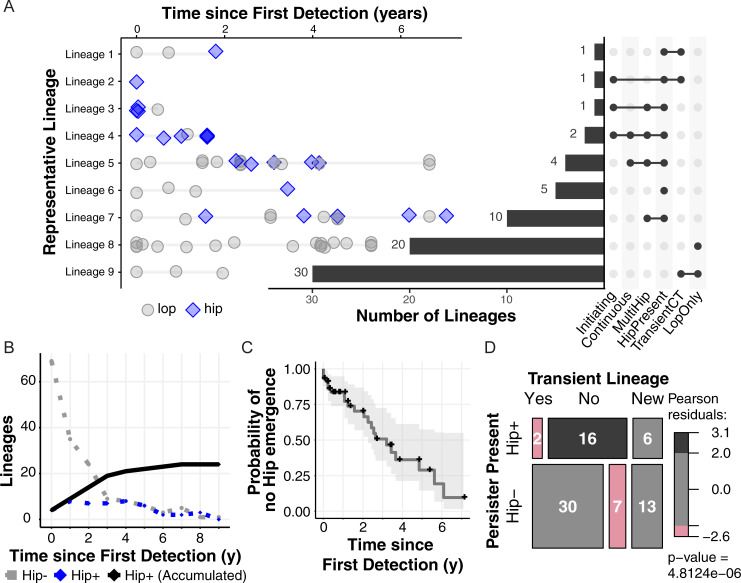
High-persister incidence patterns from a lineage-based perspective. **(A)** Lineages were classed according to several nested characteristics: transient versus non-transient lineages, Hip presence, presence of multiple Hips, continuous periods of isolated Hips, and lineage-initiating Hips. Lineages representing each combination of traits are shown on the left (Hip blue diamonds, Lop grey circles), while characteristic sets are identified and enumerated for the entire collection on the right. **(B)** The continuous lineage count of Hip- lineages (grey circles) versus Hip+ lineages (blue diamonds) for the prior year of colonization is plotted, while the accumulating count of Hip+ lineages from time 0 is shown by black diamonds. **(C)** A survival curve shows the probability of no Hip emergence over time since first detection of a lineage, based on time from first isolate to first Hip isolate for each lineage. The step function is shown in dark gray with confidence band in light gray. Lineages which never produce a Hip isolate are censored at time of last isolate (black crosses). **(D)** Transient lineages (lineages of shorter than 2 years duration, less than 50% of total patient infection length, and which are followed by the appearance of a new lineage) are significantly associated with lineages lacking Hips (Hip-), while non-transient lineages are associated with the presence of Hips based on Pearson's chi-squared test (via a mosaic plot visualizing a multi-way contingency table). Transience-unclassifiable lineages of shorter than 2 years’ duration at the end of a patient’s collection period are shown for context (‘New’).

To illustrate the range of persister dynamics we observe, we grouped each lineage by an array of descriptors. The lineage descriptors include Hip presence versus absence (Hip+ vs Hip-), presence of multiple Hips in the same lineage (MultiHip), transience of the lineage (whether it appears for less than 2 years, less than half the length of a patient’s infection and is afterwards replaced by another lineage), continuity of Hip variants (whether Hips are present for at least 3 sampling dates in a row), and whether a Hip variant initiates the lineage. Fig 3A shows the ordered distribution of the lineages in 10 different groups based on descriptor sets, illustrating the diversity of lineage Hip dynamics. We see that: 1) 24 of 74 lineages are Hip+, 2), 17 of 24 Hip+ lineages produce multiple Hip isolates, 3) 30 of 50 Hip- lineages are transient, while 2 Hip+ lineages are transient, 4) 6 lineages exhibit continuous periods of Hip variants, and 5) 4 lineages have initiating Hip+ variants. Thus, the fraction (18.9%, Fig 1D) of total isolates with a Hip phenotype appears to be distributed over a subset of lineages (32.4%) in both stable (continuous) and stochastic patterns of incidence (Fig 3A), rather than present in every evolving lineage.

### Hip variants accumulate over colonization time and occur rarely in transient lineages

We summarized the incidence of Hip variants over each lineage’s time of colonization in Fig 3B. Here we plot the continuous counts of patients exhibiting the Hip phenotype (Hip+) versus no Hip presence (Hip-) within the previous year (dashed lines) as well as the accumulation of lineages that have exhibited a Hip variant at least once by a certain age of colonization (solid line). This illustrates the number of lineages assessed at a given colonization age and the increasing fraction of Hip+ lineages over time, respectively. Overall, Hip variants affect 24 lineages (Fig 3B) and 56% of the patients in our study cohort (S3 Fig) by the end of the study period. Fig 3C shows the probability of no Hip emergence (i.e. lineage survival without Hip) over time since the lineage was first detected, which shows the impact of lineages without Hips disappearing (the black crosses indicate where they have been censored at their last collected isolate date) in addition to the step decreases associated with the date of first Hip in Hip+ lineages. We investigated this phenomenon further by evaluating the relationship between lineage transience and Hip presence. We first mark the lineages present for less than 2 years in a patient at the end of their monitoring period as ‘New’ lineages since we cannot determine transience without additional samples. Of the 55 remaining lineages, non-transient lineages are significantly associated with the Hip+ lineage status (Fig 3D). Thus, a given patient often has multiple infecting lineages, but the Hip- lineages are much more likely to disappear over the course of infection.

### Hip variants associate with frequent antibiotic treatment and eradication failure in patients

The few persister studies that incorporate clinical data have shown the emergence of Hips after antibiotic treatment, with a recent work showing how multi-drug treatment induces tolerance before resistance in two patients infected with methicillin-resistant *Staphylococcus aureus* [31]. Here, we have the opportunity to analyze the relationship between treatment and the Hip phenotype within a substantial patient population under similar treatment regimens. At the Copenhagen CF Center, patients are treated aggressively with a broad range of antibiotics, including 9 classes of *P*. *aeruginosa* targeting treatments (aminoglycosides, carbapenems, cephalosporins, fluoroquinolones, macrolides, monobactams, penicillins, and penicillins paired with beta-lactamases). Patients are regularly prescribed combination therapy using fluoroquinolones, polymyxins, and aminoglycosides (ciprofloxacin-colistin or ciprofloxacin-tobramycin), and treatment is adjusted and recorded at ~monthly clinic visits.

**Fig 4:**
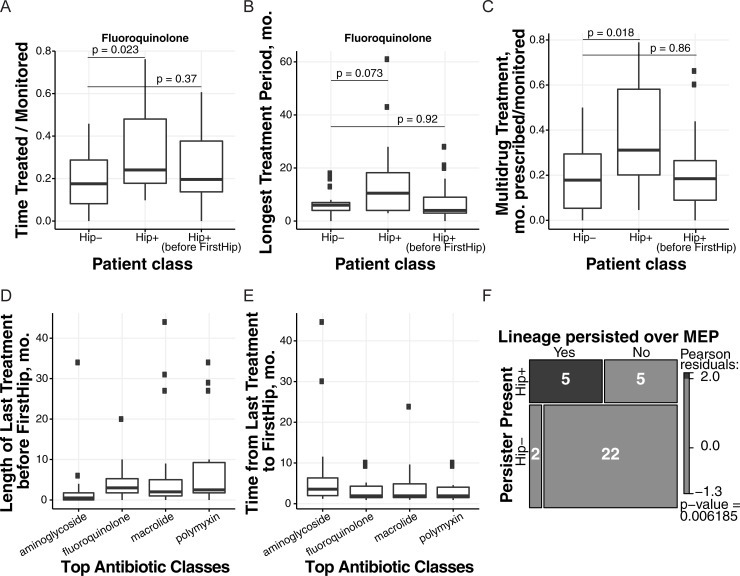
High-persister incidence versus patient treatment. **(A-C)** Differences in treatment between Hip- patients, Hip+ patients, and Hip+ patients prior to their first Hip were evaluated using clinical records of treatment with antibiotics active against *Pseudomonas aeruginosa*. We focus on fluoroquinolone treatments, which includes ciprofloxacin (used regularly for nearly all patients) and ofloxacin. (**A**) The degree of fluoroquinolone treatment was determined by summing the length of treatment periods and dividing by the length of each patient’s monitoring period (to normalize data across patients). (**B**) The longest continuous treatment period with ciprofloxacin was determined by finding the longest continuous span of months where a drug was prescribed. (**C**) The number of months where 3 or more *P*. *aeruginosa*-targeting antibiotics were prescribed divided by monitoring timespan was used to assess elevated drug use. (**D-E**) The top 4 *P*. *aeruginosa*-targeting antibiotic classes prescribed to the patients include aminoglycosides (top drug: tobramycin), fluoroquinolones (top drug: ciprofloxacin), macrolides (top drug: azithromycin) and polymyxins (top drug: colistin). We evaluated the months these drugs were prescribed in Hip+ patients in the period before their first Hip was isolated. (**D**) We evaluated the length of the last treatment (in months prescribed) preceding the month of Hip emergence. (**E**). The time between this last treatment month to the month of Hip emergence (a value of 0 means the drug was used in a patient the month prior to Hip emergence) is shown. (**F**) Hip presence is evaluated in the lineages of 20 patients assessed previously in a study of strain persistence over their maximum ‘eradication’ period, the MEP (at least 6 months of *P*. *aeruginosa*-negative sputum) via a mosaic plot. The majority of lineages which persisted through apparent eradication periods are Hip+. Statistics: Panels A-C show boxplots for Hip+ versus Hip- patients (Hip- N = 17, Hip+ N = 22) where difference in population distributions is tested via an unpaired Wilcoxon test of the means for each Hip+ population versus the Hip- population. Panels D-E show boxplots for data from all Hip+ patients (N = 22). Panel F evaluates associations between groups based on Pearson's chi-squared test.

Here, we compare Hip incidence with a patient’s length of treatment time as well as counts of the months a given drug is prescribed (collapsing IV versus per-oral versus inhaled treatment together for expediency). We assess the data in a patient-specific manner from one year prior to their first isolate assessed in this study to their last isolate (Hip+ and Hip- patient categories) as well as from one year prior to their first isolate to their first Hip isolation date (Hip+ before FirstHip, truncating the Hip+ patient records). Fig 4A and 4B shows different metrics of fluoroquinolone treatment for these groups: Total time treated with a fluoroquinolone normalized by patient monitoring timespan and the longest continuous treatment period (by months in a row that a drug is prescribed). These data show that while Hip+ patients receive on average much more fluoroquinolone treatment than Hip- patients, assessing Hip- patients versus Hip+ patients before their first Hip shows similar antibiotic usage in these more directly comparable timeframes (Fig 4A and 4B, means compared by unpaired Wilcoxon test). Even non-normalized total time of fluoroquinolone treatment is not significantly different between Hip- patients versus Hip+ patients before their first Hip. Furthermore, an assessment of multidrug treatment, which we view as treatment with at least 3 PA targeting drugs within the same month given that two drug classes are already commonly used together, also shows the same trend (Fig 4C, means compared by unpaired Wilcoxon test). Therefore, Hips emerge in about half of our study patients, and all of these patients are being given similar treatment rather than any extra treatment applied to the Hip+ population before Hip emergence. However, Hip+ patients do on average undergo more aggressive treatment than Hip- patients when their entire monitoring period is assessed (Fig 4A, 4B, and 4C).

For the most commonly prescribed drug classes in our cohort, we look further into the Hip+ dataset before the first Hip is isolated (Fig 4D and 4E). First, we see that a few patients have not been treated with fluoroquinolone at all prior to their first Hip (Fig 4D). The median length of the fluoroquinolone treatment directly before the first Hip is 3 months where the antibiotic was prescribed, but this value is not effectively different than the median for macrolides or polymyxins (2 and 2.5 months, respectively, Fig 4D). We show in Fig 4E that the median of time between the last fluoroquinolone treatment and first Hip isolation (N = 20 of 22 Hip+ patients) is 2 months, and this trend again also holds true for macrolides (N = 21 of 22 Hip+ patients) and polymyxins (N = 20 of 22 Hip+ patients). Our first Hips therefore often arise after a recent treatment for multiple months with a panel of PA targeting drugs. However, 15 of 17 Hip- patients also receive similar multi-drug treatments. Ultimately, we cannot conclude that fluoroquinolone treatment, rather than a panel of drugs, is specifically driving the development of and selection for Hips identified via a ciprofloxacin screen, and we again do not observe evidence for multi-drug treatment inducing the first Hip isolate.

While the relationship between antibiotic treatment and the Hip phenotype is complex, we see more definitive patterns in an assessment of treatment failure which connects our analysis of Hip presence in non-transient lineages to a clinically relevant diagnostic (Fig 3C). A recent study of strain eradication, defined in the Copenhagen clinic as a failure to culture *P*. *aeruginosa* from a six month period of routine sputum sampling, evaluated whether strains in fact persisted over the longest supposed eradication period in a patient’s clinical history (the maximum eradication period, or MEP). Historically, eradication periods are viewed as markers of treatment success, and strains that recur after eradication periods are assumed to represent new infections, but our study showed that over 40% of patients had a strain persist over their maximum eradication period[39]. As these adapted strains are likely less susceptible to treatment than a new infection, this misdiagnosis of eradication is of great concern for the clinic and should influence treatment decisions. We assessed whether strains that persisted versus were eradicated over the MEP produced Hip isolates in the 20 patients which were also included in this study. As Fig 4F shows via a mosaic plot, 5 of 7 strains that persisted in 7 respective patients also produced Hip isolates. Four of these strains produced a Hip before the MEP within the representative isolates which we assayed here, and one produced a Hip before a second ‘eradication’ period nearly equal to the length of the MEP (.86 versus .89 years ‘eradicated’). In contrast, only 5 of 22 strains that disappeared from patient lungs prior to a true MEP actually produced Hips. This association supports the theory that the Hip phenotype contributes to treatment failure, and provides an explanation for why the clinic’s culture-based eradication metric and associated treatment guidelines are insufficient for many infections.

### Hip variants show increased fitness in patient-similar biofilms

**Fig 5:**
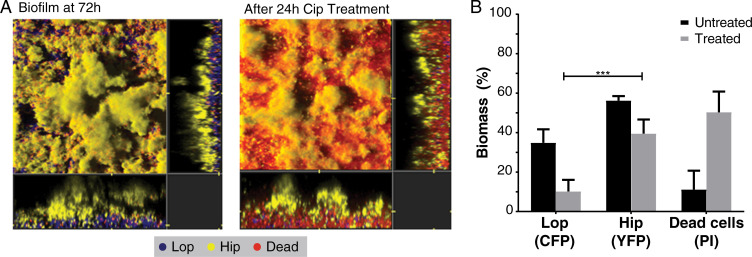
Fitness comparison of Lop and Hip isolates in biofilm conditions. **(A)** A representative Lop and Hip isolate with similar characteristics (Lop: cip MIC 1.0 μg/ml; growth rate 0.62 hr^-1^; time since first detection 4.28 years. Hip: cip MIC 0.75 μg/ml; growth rate 0.57 hr^-1^; time since first detection 5.49 years) were differentially tagged with CFP (Lop) or YFP (Hip). Tagged isolates were cocultured and allowed to form biofilms in a flow-cell model for 72 hours. Mixed biofilms were treated for 24 hours with ciprofloxacin (4 μg/ml). Propidium iodine (PI) was added to visualise dead cells (red). **(B)** Biomass was quantified for each population. Significant differences in biomass following treatment were determined using unpaired t-test (*** p <0.001).

We directly tested whether Hip isolates are able to survive antibiotic treatment better than Lop isolates with similar antibiotic susceptibilities and growth properties in more complex conditions. We simulated antibiotic treatment of CF patients in a recently developed biofilm Pharmacokinetic/Pharmacodynamic (PK/PD) system, in which the bacteria are challenged with antibiotics in much the same way as in patients [40]. We chose this model because *P*. *aeruginosa* often appears as structured populations in lungs of CF patients [41], because biofilms have been shown to harbor increased levels of persister cells [42], and because our model mimics the bacterial exposure to ciprofloxacin treatment as described for CF patients [40,43]. The isolates that were chosen shared similar 1) time since the strain’s first detection in the CF lungs, 2) MIC values for ciprofloxacin, 3) growth rates, and 4) belong to the same clone type. The Hip/Lop pair was differentially tagged with yellow fluorescent protein, YFP (Hip), or cyan fluorescent protein, CFP (Lop). After checking that both strains formed biofilms with comparable biomasses in the flow-cell system, Hip and Lop cells were mixed 1:1 and allowed to form a mixed biofilm. Representative images of the Hip/Lop biofilms are shown before and after treatment with ciprofloxacin (Fig 5A).

The majority of Lop bacteria were located close to the glass substratum with the Hip population proliferating at the external surface of the biofilm, facing the liquid flow. The addition of ciprofloxacin preferentially killed the Lop population leaving the Hip population relatively unaffected by the antibiotic. COMSTAT analysis confirmed this changed population structure after ciprofloxacin addition (Fig 5B). This documentation of a Hip associated fitness increase in an antibiotic containing environment is also novel as it has been shown previously that ciprofloxacin treatment of flow-cell biofilms preferentially kills the surface sub-populations of micro-colonies [44]–yet the Hip cells on the colony surfaces survive much better than the internal Lop bacteria under treatment with ciprofloxacin.

In summary, we find that despite variable incidence patterns, half of patients are infected by Hip+ lineages, and these lineages have a significant persistence advantage in comparison to Hip- lineages over time. Via both an analysis of our patients’ treatment histories as well as a competition experiment using an *in vitro* model replicating dynamic treatment with ciprofloxacin, we provide comprehensive support for the theory that the Hip phenotype contributes to a fitness increase in antibiotic-treated patients.

## Discussion

The objectives of this investigation have been to 1) determine the clinical importance of *P*. *aeruginosa* persister populations in antibiotic treated CF patients, 2) search for variant lineages with increased levels of antibiotic tolerant subpopulations (Hip) during treatment, and 3) define the Hip phenotype in relation to resistance and previously described persister cells. The background is an increasing awareness of antibiotic treatment failure in the CF clinics, which cannot be ascribed to antibiotic resistance development in the early years of infection.

We have mapped the prevalence of high-persisters in a large, aligned cohort of patients under intensive antibiotic treatment for a 10 year period [24,43]. Of 460 *P*. *aeruginosa* isolates from the airways of 39 young CF patients (74 lineages in total), 18.9% of the isolates were scored as robustly persisting Hip using a high-throughput screening approach to assay persistence against ciprofloxacin (Fig 1). We show that the isolates display different levels of persisters, in accordance with the variance previously found between species and within strains [5,45,46]. Most adaptive changes occur during the first few years of colonization [20,47], which matches our objective of searching for signs of increased fitness of Hip variants in patients treated continuously with antibiotics. We show that in a young CF patient cohort impacted by early longitudinal colonization by *P*. *aeruginosa* strains, Hip variants were sampled from 56% of the patients (N = 22) during a 10-year observation window. Our analysis is a new and useful comparative baseline for developing effective surveillance, impact assessment, and eventual control of the persister phenotype in the clinic.

Multiple relationships between the Hip phenotype and other phenotypic traits such as growth rate and antibiotic resistance have been suggested in the literature. While some studies point out that there is no correlation between the mean growth rates of isolates and the observed Hip phenotype [48–50], reduced growth rates have been associated with high persister phenotypes in *E*. *coli* [32]. A recent study in *Salmonella enterica* further supports that slow growth (regardless of mechanism) promotes the high-persister phenotype [35]. Drug-tolerant cells have also been proposed to facilitate evolution of true antibiotic resistance in *E*. *coli*, where intermittent antibiotic exposure of a batch culture selected for mutant clones harboring tolerance mutations that increased the growth lag-time, during which tolerance to killing by ampicillin selected for MIC-increasing mutations [30]. Early isolates of a *S*. *aureus* lineage from a patient treated with antibiotics also acquired tolerance, and subsequently resistance [31]. Though *P*. *aeruginosa* in the CF lung is also exposed to fluctuating concentrations of antibiotic, we see that our lineages which produce Hips are also often producing isolates with reduced growth rate and/or ciprofloxacin resistance over time (Fig 2). The stringently defined persister phenotype described in the current study is often observed after one of these trait adaptations if not both. In summary, our results suggest that the Hip phenotype is an early and common advantageous adaptation [20] arising stochastically in infected patients alongside other beneficial adaptations.

Many studies have shown the increased survival of persister cells under antibiotic treatment [32,51] and then screened for genetic determinants of persistence, but few have evaluated the fitness of Hip versus Lop variants in direct competition experiments [45]. In our study, we tested a single Lop/Hip pair of isolates matched by genotype, phenotype, and colonization age in order to characterize the selective advantage of the Hip phenotype in a biofilm under treatment-replicating antibiotic exposure [40]. We show that Hip cells survived ciprofloxacin treatment far better than Lop isolates. It is also striking that the *in vitro* biofilm fitness assessment shows efficient elimination of the Lop strain in the presence of ciprofloxacin, whereas Hip variants often coexist with Lop variants *in vivo* (Fig 4A). This suggests that in the patient, direct competition is likely impacted by uneven distribution of antibiotics, many separate regional niches and selection forces, and influence of the host [52]. Our analysis is an illustration of a Hip associated fitness increase in the presence of antibiotics, limited to one Lop/Hip pairing, and additional experimentation is warranted to determine the broader applicability of the current findings.

In many ways, investigations of the genetic underpinnings of high-persisters have been performed analogously to studies of antibiotic resistance, i.e. it was expected that a relatively limited set of genes defines the phenotype. In a study of urinary tract infection *E*. *coli* isolates, a gain-of-function mutation in the HipA toxin was commonly observed [23]. In contrast, a lack of common targeted genes in a small collection of clinical Hip strains of *Mycobacterium tuberculosis* suggested utilization of multiple genetic pathways [53]. Working at a much larger collection scale in a faster adapting organism, we do not see enrichment of mutations which previously have been associated with Hip phenotypes. Furthermore, the enrichment level of identified mutated genes is low, with few targets associated with a high number of Hip+ lineages. We therefore conclude that a Hip phenotype may derive from a diverse array of accumulating genetic changes, and it is likely that more than one mutation often determines the persister level in the respective bacterial populations[54,55]. We also cannot know whether our enriched mutations are gain or loss of function mutations, nor can we be sure of their effects without reversion in their own strain background rather than a more friendly lab strain; as other traits clearly may be mutational targets versus our persister phenotype (Fig 2), we did not pursue this. Our results likely reflect the multiple and dynamic selection pressures *in vivo*, which challenge Hip variants in antibiotic-treated populations very differently than those assessed in steady state *in vitro* conditions with only one selective force.

Our comprehensive integration of clinical data, both treatment records for 39 patients and clinical metrics of eradication failure for 20 patients, provides novel context for Hip formation and fitness contributions in the patient environment at a new scale. Antibiotic treatment is aggressive and diverse in our patient population, but when evaluating comparable Hip-free time periods for Hip+ and Hip- patients, we do not observe elevated treatment with fluoroquinolone or other PA targeting drugs that suggests a specific regimen consistently selects for Hips. However, heavier treatment follows the emergence of a Hip isolate in Hip+ patients compared to Hip- patients, and Hip+ lineages are more likely to persist over long ‘eradication’ periods than Hip- lineages. We interpret these phenomena as due to a likely intensification of treatment failure after Hip emergence which necessitates ever more aggressive (yet failing) measures against the persisting, Hip producing strain in comparison to Hip- patients. We demonstrate this association between Hip presence and treatment failure in a worst-case scenario, where patients are treated intensively. Ultimately, identifying altered treatment regimens that might prevent Hip emergence is still difficult, given that Hip- and Hip+ patients undergo such similar treatment prior to the first Hip isolate. However, monitoring the emergence of Hip isolates appears promising as a prognostic for strain persistence in the clinic, and could also support development of treatment regimens which actively target persisters or reduce the fitness benefit of this phenotype in patients once it emerges.

In summary, we have shown that Hip variants of *P*. *aeruginosa* emerge frequently in young CF patients, and our results provide a first window into the evolving landscape of persistence across a whole patient cohort. As pathogens increase their fitness in patients over time, they clearly evolve a high-persister phenotype as an important component in their survival repertoire and can do so from the earliest stages of infection. It is still premature to conclude that the high-persister phenotype described here differs from what has been identified as Hip in *in vitro* experimental conditions, but we consistently find a much broader bacterial repertoire for survival in patient lungs. Notably, there seems to be no direct link between presence of the relevant antibiotic (ciprofloxacin) and Hip appearance, and Hip variants do not seem to be mutated in genes previously found from *in vitro* experiments to associate with Hip or in any strongly conserved genetic route. We suggest that the difference in complexity of selection pressures when comparing *in vitro* and *in vivo* environmental conditions results in highly different evolutionary trajectories, and that a Hip phenotype may be selected for by other forces than presence of antibiotics. With our investigation, we provide an important platform for broader clinically based studies and contribute important new context for monitoring and one day hopefully preventing the high persister phenotype in the clinic.

## Materials and methods

### Ethics statement

All patients (or their legal guardians) consented to the collection of sputum samples as a part of routine treatment at the Cystic Fibrosis Center at Rigshospitalet, Copenhagen which includes regular culture, biobanking and clone typing of bacteriological isolates. All samples and data used in this study (patient origin of bacterial isolate and associated clinical metadata) were pseudonymized according to local ethics guidelines, so further patient consent was not required. The local ethics committee at the Capital Region of Denmark (Region Hovedstaden) approved the use of the stored *P*. *aeruginosa* isolates (registration number H-4-2015-FSP). The Danish Agency for Patient Safety (registration number 31-1521-428) approved the analysis of pseudonymized microbiological records from the clinical microbiology database (MADS) and treatment data (journal number 2008-41-2682). These data were provided by the management of the Department of Clinical Microbiology at Rigshospitalet who also approved of the study. We confirm that all methods were performed in accordance with the relevant guidelines and regulations.

### Strain collection

In total, we analyzed 460 *P*. *aeruginosa* airway isolates from young CF patients followed at the Copenhagen CF-clinic at Rigshospitalet (S1 Dataset). The local ethics committee at the Capital Region of Denmark (Region Hovedstaden) approved the use of the stored *P*. *aeruginosa* isolates: registration number H-4-2015-FSP. Phenotyping data for 434 isolates of this strain collection (growth rate in LB, adhesion in LB, and ciprofloxacin MIC) have been previously published [20]. We include additional MIC measurements and expand the complete trait dataset to 446 isolates. All available trait data is provided in S1 Dataset along with the persister classification of each isolate and descriptive data.

Of the 460 isolates examined in the study, 403 isolates from 32 patients were described previously in Marvig et. al. [24] and the remaining isolates were taken from seven previously undescribed patients. The isolates were collected and stored at the Department of Clinical Microbiology at Rigshospitalet, Copenhagen, Denmark, between 2002 and 2014. Of the patients included in this study, 35.9% were diagnosed as chronically infected with *P*. *aeruginosa* by the end of the study period. We defined chronicity based on the Copenhagen CF Centre definition, whereby either *P*. *aeruginosa* has been detected in six consecutive monthly sputum samples or fewer consecutive sputum samples combined with observation of two or more *P*. *aeruginosa*-specific precipitating antibodies [43,56]. Intermittently colonized patients were defined as patients where at least one isolate of *P*. *aeruginosa* is detected, and normal levels of precipitating antibiotics against *P*. *aeruginosa* were observed.

Pseudonymized patient treatment data was assembled using records kept by the Copenhagen CF Centre administration for the following PA-targeting antibiotics: ciprofloxacin, colistin, tobramycin, azithromycin, meropenem, piperacillin/tazobactam, clarithromycin, ceftazidime, aztreonam, ofloxacin, amikacin, imipenem, and piperacillin. Per-oral, inhaled and intravenous (IV) treatments of these antibiotics were collapsed into broader antibiotic classes (with focus on the length of time drugs from a given class were applied rather than attempting a more complex estimate of combined antibiotic dosing). Treatment length (originally recorded as weeks of treatment in the CF Centre database) and count of months a given drug was prescribed (whether a 2 week IV course or a month of inhalation) were then used as treatment metrics. For example, 2 weeks of tobramycin IV therapy one month might be followed by a full month of inhaled treatment, which we allow as essentially continuous tobramycin therapy when looking for a stressful period of treatment that might select for the Hip phenotype. Records for the Hip+ and Hip- patient populations ran from 1 year prior to each patient’s first isolate included in our study to their last isolate included in our study (termed patient-specific monitoring periods). A subset of the Hip+ treatment data, from 1 year prior to each patient’s first isolate to the date of their first Hip isolate, was also evaluated. The Danish Agency for Patient Safety and the administrators of the Copenhagen Cystic Fibrosis Centre approved the analysis of treatment data: registration number 31-1521-428.

### High-throughput screening for Hip mutants

To determine the frequency at which *P*. *aeruginosa* Hip mutants emerge in CF patients, we screened 460 isolates for the ‘persister’ phenotype against ciprofloxacin. Stock 96-well microtiter plates containing 4 technical replicates of each isolate stored in glycerol (25% v/v) were prepared and stored at -80°C. Using a 96-well spot replicator, bacteria were transferred from the stock plates into sterile 96-well microtiter plates containing 150 μl of Lysogeny Broth (LB) media. Plates were incubated statically for 48 hours at 37°C until the bacteria reached the stationary phase of growth. To determine the initial viability of bacteria in each well, the replicator was used to spot bacteria onto LB agar plates. Subsequently, 100 μg/ml of ciprofloxacin was added to each well and the microtiter plates were incubated statically for a further 20–24 hours at 37°C. Serial dilutions were performed in 96 well microtiter plates containing 0.9% NaCl using an automated fluid handling robot (Viaflo3844/ Integra Biosciences AG). Each dilution was spotted onto LB agar plates using the replicator. Approximately 0.5μL was transferred using the replicator, resulting in a limit for detection of 2x10^3^CFU/ml. Plates were incubated at 37°C for at least 48 hours. The growth of the bacteria was then compared by counting colonies whenever possible and visually inspecting growth on the plates before and after antibiotic treatment. Experiments were performed in duplicate.

### Persister assay validation

Time-kill experiments were performed for six isolates from the same lineage (3 Hip and 3 Lop). *P*. *aeruginosa* were inoculated in 3 ml of LB media in 14 ml culture tubes and incubated for 48 hours at 37°C with shaking at 250 rpm. Following incubation, each culture was serially diluted using sterile 0.9% NaCl, plated onto LB agar and incubated at 37°C to determine the initial colony forming units (CFU). The remaining culture was treated with either 10 μg/ml or 100 μg/ml of ciprofloxacin and incubated at 37°C with shaking. Cultures were washed and diluted in sterile 0.9% NaCl, then spot plated onto LB agar 6 and 24 hours after the addition of antibiotic. Plates were incubated for 24 hours at 37°C. Bacteria survival was measured by counting CFU per ml. To determine if surviving bacteria (i.e. persisters) produce the same killing kinetics as the initial isolate, three independent colonies were picked following 24 hours of ciprofloxacin treatment (100 μg/ml) and then persister assays were repeated as described above. For an additional 19 isolates, the same validation experiment was performed, however cultures were only plated out after 24 hours, which was within the ‘persister plateau’. Persister validation assays were performed using at least three biological replicates.

### Phenotype screening

The same frozen library of isolates used in the persister screening was also used for assay of minimum inhibitory concentrations (MICs), bacterial growth, and adhesion as described below and in Bartell et al [20]. MICs for ciprofloxacin were determined using E-test methodology according to the manufacturer’s recommendations (Liofilchem, Italy). To assay growth rate, bacteria were replicated from frozen plates into 96 well plates containing 150μL of LB medium, and incubated for 20 hours at 37°C with constant shaking. OD 630 nm measurements were taken every 20 minutes using a microplate reader (Holm & Halby, Copenhagen, Denmark/Synergy H1). Generation times (Td) were determined on the best-fit line of a minimum of 3 timepoints during exponential growth of the bacterial isolate. Growth rates (hr^-1^) were calculated using the formula log (2)/ Td using semi-automated code described in Bartell et al [20]. Adhesion was measured via attachment assays in 96-well plates using NUNC peg lids and 96 well plates with 150μl Luria broth medium. OD_600nm_ was measured after incubation for 20 hours at 37°C and subsequently, a “washing microtiter plate” with 180μl PBS was used to wash the peg lids and remove non-adhering cells. After transfer of the peg lids to a microtiter plate containing 160μl 0.01% crystal violet (CV), they were left to stain for 15 min. To remove unbound crystal violet, the lids were then washed again three times in three individual “washing microtiter plates” with 180μl PBS. Adhesion was measured by detaching adhering CV stained cells through washing the peg lids in a microtiter plate containing 180μl 99% ethanol. An ELISA reader was then used to measure the CV density at OD_590nm_. (Microtiter plates were bought at Fisher Scientific, NUNC Cat no. 167008, peg lids cat no. 445497).

### Pharmacokinetic/Pharmacodynamic (PK/PD) flow chamber biofilm model

For fitness experiments, we used a PK/PD biofilm model system combined with confocal laser-scanning microscopy. This system simulates the changing antibiotic concentrations in CF patients during intravenous dosing in addition to retaining a similar profile of antibiotic decay as the one taking place in CF patients [40]. First, Hip and Lop isolates were differentially tagged with a yellow fluorescent protein (YFP) or cyan fluorescent protein (CFP) respectively [16]. Flow chambers were inoculated with a 1:1 mixture of Hip and Lop bacteria (each isolate had an initial OD_600_ of 0.5). Bacteria were incubated for one hour at 30°C, then nutrient flow was applied to each chamber (40x diluted LB at a rate of 20 ml/h using a Watson Marlow 205S peristaltic pump). Biofilms were allowed to form for 72 hours, at which point flow was stopped and medium containing ciprofloxacin was added. Peak ciprofloxacin concentrations were calculated to be 4 mg/L based on PK parameters generated from healthy patients and CF patients [57]. The medium was pumped from the dilution flask through the antibiotic flask to the flow chambers at a constant rate calculated to mimic the elimination rate constant of the antibiotic for 24 hrs. A confocal laser-scanning microscope (Zeiss LSM 510) equipped with an argon/krypton laser and detectors was used to monitor YFP (excitation 514 nm, emission 530 nm), CFP (excitation 458 nm, emission 490 nm), and dead cells (propidium iodine, excitation 543 nm, emission 565 nm). Multichannel simulated fluorescent projections (SFPs) and sections through the biofilms were generated using Imaris software (Bitplane AG, Switzerland). The images were later analyzed using COMSTAT [58]. The PK/PD biofilm experiments were performed using two independent Hip/Lop isolate pairs. Pairs were taken from the same patient at a similar time since first detection and had similar growth rates and ciprofloxacin MICs (Fig 5 legend). The data presented are from 2 biological experiments with 4 independent images taken from each experiment.

### Lineage-based genetic analysis

To generate a list of mutated genes associated with the Hip phenotype, we used previously generated whole-genome sequencing data and variant calling filtered to obtain nonsynonymous mutations that had accumulated within a lineage after the first isolate [24] to evaluate differential mutation patterns for Lop and Hip variants for 403 sequenced isolates. In this filtering process, we also removed mutations associated with any known ‘hypermutator’ isolates based on a mutation in *mutS* or *mutL* to avoid the influence of high random mutation in these isolates on the analysis. To identify genes that were mutated more than would have been expected by drift/random mutation while accounting for lineage-based mutation accumulation over time, we adapted a statistical analysis of the relative mutation enrichment by lineage. After separating Lop and Hip variants, we compared the mutated-gene lineage enrichment ratios for each group—the number of lineages with observed mutation(s) in a given gene divided by the number of lineages expected to have mutations in that gene according to random mutation. This enrichment metric was obtained as follows for each group: we determined the observed number of lineages mutated (sum-obs) in each gene. Then we estimated the average number of lineages (avg-exp) that would have been mutated in each gene if mutations were spread out randomly over the PAO1 genome. Using a random-roulette algorithm, the number of genes that were observed to be mutated in a given lineage was spread out over the PAO1 genome for 1000 iterations, providing a *m*_gene_ by *n*_iteration_ matrix of randomly mutated gene profiles for each lineage. For the same iteration *n* across all lineages, it was noted whether a given gene was mutated. This allowed us to determine an average number of lineages expected to be mutated over 1000 iterations. If a gene was hit by chance more than once in a single iteration, this would still only be denoted as one hit; this is in alignment with our observed mutation assessment, where multiple isolates could be hit in the same gene but we only noted whether or not the lineage was hit by unique mutations in the specific gene. After obtaining the relative enrichment by lineage, a Poisson distribution was used to calculate the probability of the observed given random drift (expected). We also divided the lineage enrichment metric for genes mutated in Hip variants by that for Lop variants to obtain a lineage enrichment ratio to identify targeted genes particularly impactful in the evolution of the Hip population.

### Data analysis and statistics

Analyses were conducted in RStudio v. 1.0.143 and R v. 3.4.0 with visualization package ggplot2 v. 3.0.0. Lineage set analysis was performed using UpSetR v. 1.3.3 in R [59]. Principal component analysis was performed in R using ‘prcomp’ with centered and scaled phenotype data (S1 Dataset). Mosaic plots (visualizing multi-way contingency tables) showing the association between two variables via the conditional relative frequency and significant associations based on a Pearson X^2^ test were created using vcd v. 1.4–4 in R [60].

## Supporting information

S1 Fig Surviving colony forming units of Hip strains in persister assays using different ciprofloxacin concentrations. *P*. *aeruginosa* Hip isolates from the patient examined in Fig 1C were treated with 10 versus 100 μg/ml of ciprofloxacin for 24 hours, then plated on agar for surviving CFU determination. Each isolate was tested independently 3 times. The data are represented by the mean and SD.
(EPS)

S2 Fig Correlation between persister score and colony forming units following treatment with ciprofloxacin. *P*. *aeruginosa* isolates representing each of the scores possible from the high-throughput screen (0–4) were treated with 100 μg/ml of ciprofloxacin for 24 hours, then plated on agar for surviving CFU determination. Laboratory strain PAO1 was included as a control. Each isolate was tested independently at least 4 times. The data are represented by the mean and SEM.
(TIF)

S3 Fig Hip accumulation in patients. Hip^-^ (gray squares) and Hip^+^ (blue diamonds) show the continuous count of patients with Hip^-^ lineage(s) versus Hip^+^ lineage(s) for the prior year of colonization, while the accumulating count of patients with Hip^+^ lineages from time 0 is shown by black circles.
(EPS)

S1 Table Comparison of persister genes identified in previous *P*. *aeruginosa* studies to mutated genes highlighted by our lineage analysis. (XLSX)

S1 Dataset Isolate collection data, metadata, and screen results. Description of all isolates including age, genotypic, and phenotypic information used in the analysis. Specific legend included below and in dataset file.
(XLSX)

S2 Dataset Lineage-based mutation enrichment analysis for coding genes and noncoding regions. Mutated genes enriched in Hip versus Lop isolates as assessed from a convergent evolution perspective accounting for lineage adaptation. Lineage enrichment ratio was calculated by dividing lineage-based gene mutation enrichment within Hip variants by that within Lop variants for each gene. Top Hip-linked genes were selected via the following criteria: greater than 2 lineages presenting mutations in that gene in the Hip population and a lineage enrichment ratio greater than 2.
(XLSX)
