# Supplementary figures and images for: Bacterial persisters in long-term infection: Emergence and fitness in a complex host environment
Source: PLoS Pathog. 2020 Dec 14;16(12):e1009112. doi: 10.1371/journal.ppat.1009112 (PMC7769609; doi:10.1371/journal.ppat.1009112)

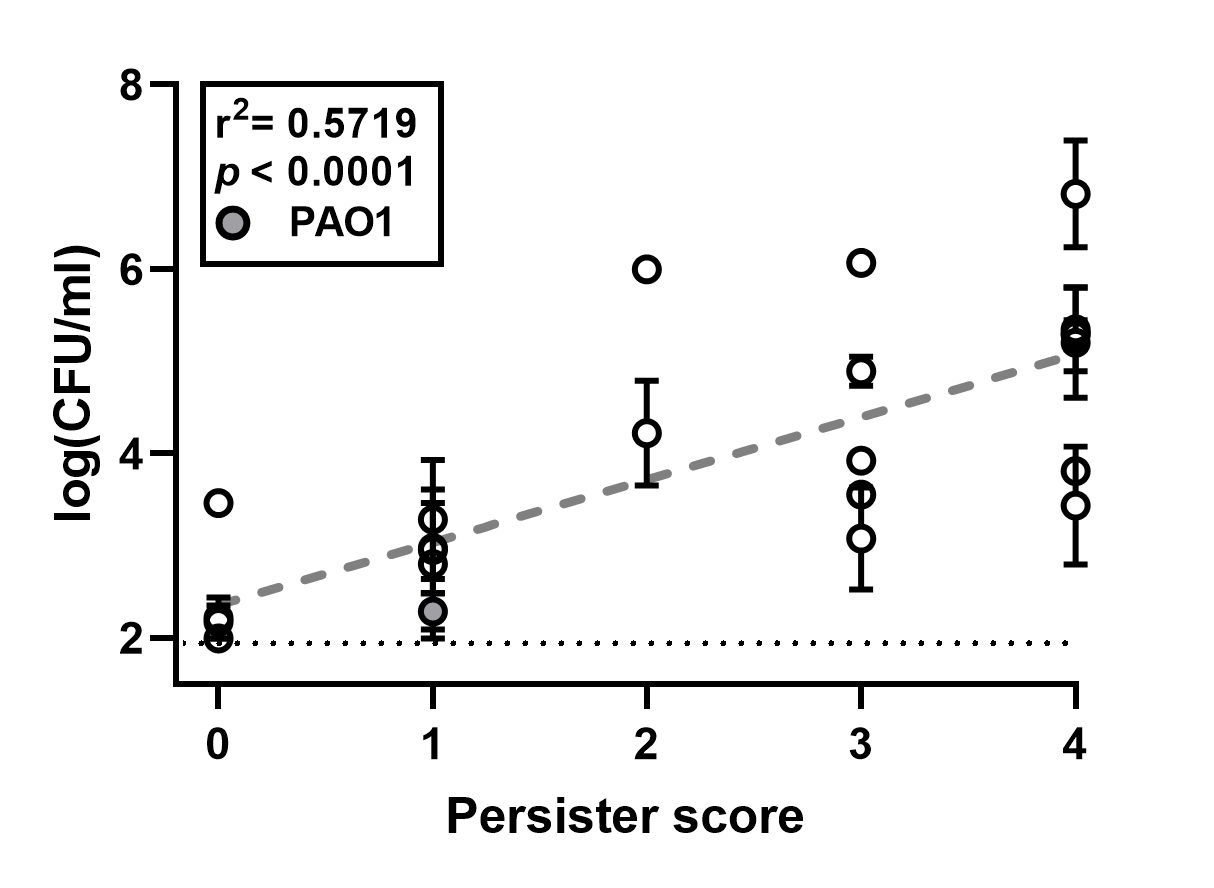

Supplement: S2 Fig — P. aeruginosa isolates representing each of the scores possible from the high-throughput screen (0–4) were treated with 100 μg/ml of ciprofloxacin for 24 hours, then plated on agar for surviving CFU determination. Laboratory strain PAO1 was included as a control. Each isolate was tested independently at least 4 times. The data are represented by the mean and SEM. (TIF) [file ppat.1009112.s002.tif]
